# Clinical, magnetic resonance imaging, surgical features and comparison of surgically treated intervertebral disc extrusion in French bulldogs

**DOI:** 10.3389/fvets.2023.1230280

**Published:** 2023-08-31

**Authors:** Guillaume Marc Albertini, Fabio Stabile, Oliver Marsh, Ane Uriarte

**Affiliations:** Neurology and Neurosurgery Department, Southfields Veterinary Specialists Part of Linnaeus Veterinary Limited, Basildon, United Kingdom

**Keywords:** French bulldog, intervertebral disc disease, intervertebral disc extrusion, MRI, surgery

## Abstract

Intervertebral disc (IVD) extrusion (IVDE) is the most reported neurological condition in French bulldogs (FBD). The aim of this study was to retrospectively evaluate neurological grade, magnetic resonance imaging (MRI), surgical findings and short-term recovery in surgically treated FBD diagnosed with IVDE referred to a single institution between January 2020 and March 2022 and to compare cervical and thoracolumbar IVDE. Data was gathered from medical records and analysed via Fischer’s Exact-Test and Kruskal Wallis-tests. Statistical significance was assumed when *p* < 0.05. Thirty-nine FBD were diagnosed with IVDE. Cervical IVDE (C-IVDE) was diagnosed in 11/39 cases; the C3-C4 IVD space was the most commonly affected site (5/11). Thoracolumbar IVDE (TL-IVDE) was diagnosed in 28 cases; the L3-L4 IVD space was the most commonly affected site (7/28). At admission, C-IVDE was significantly associated with less severe neurological grade (grade 1–2) compared to TL-IVDE (grade 2–5) (*p* < 0.001). The extruded IVD material (EIVDM) was hypointense in T2w images in 11/11C-IVDE vs. 2/28TL-IVDE, and hypointense in T1w images in 10/11C-IVDE vs. 1/28TL-IVDE. The EIVDM was hyperintense in T2w images in 0/11C-IVDE vs. 26/28TL-IVDE and iso-to-hypointense in T1w images in 1/11C-IVDE vs. 27/28TL-IVDE (*p* < 0,001). The EIVDM extended over ≥2 IVD spaces in 0/11C-IVDE vs. 19/28TL-IVDE (p < 0,001). 10/11C-IVDE underwent single ventral slot, 1/11C-IVDE underwent unilateral cervical hemilaminectomy. All TL-IVDE underwent unilateral hemilaminectomy and 19/28TL-IVDE underwent unilateral hemilaminectomy over ≥2 IVD spaces (*p* < 0,001). Haemorrhagic EIVDM was noticed intraoperatively in 1/11C-IVDE vs. 28/28TL-IVDE (*p* < 0,001). Spinal cord compression was mild in 2/11C-IVDE and 3/28TL-IVDE; moderate in 9/11C-IVDE and 16/28TL-IVDE; severe in 0/11C-IVDE and 8/28TL-IVDE. There was no spinal cord compression in 1/28TL-IVDE with foraminal IVDE. There was no statistical difference between spinal cord compression and IVDE location (*p* = 0.112). The mean time to improvement was 1.1 day in C-IVDE (range 1–2 days). 90.1% of C-IVDE improved within the first 24 h. The mean time to improvement was 2.1 days in TL-IVDE (range from 1 day to 4 days). All dogs that did not improve (5/39) were grade 5 TL-IVDEs at presentation. In FBD, TL-IVDE tended to cause higher grade of neurological dysfunction, tended to result in compression of neural structures over multiple IVD spaces and required more extensive surgical treatment than C-IVDE.

## Introduction

Chondrodystrophic breeds include, along with others, Dachshunds, Beagles, Basset Hound, Cocker Spaniels, Pembroke Welsh Corgi and French Bulldogs (FBD) (1, 2). Chondrodystrophic breeds have features of altered endochondral ossification with shortened long bones suspected related to FGF4 retrogene insertion on chromosome 12 (3). These breeds are predisposed to intervertebral disc extrusion (IVDE) (3). French bulldogs have shown a recent increase in popularity in the United Kingdom with 54,074 registrations to the Kennel Club in 2021 (4). In the FBD breed, the most reported neurological condition is IVDE (5). IVDE is used to describe the displacement of nucleus pulposus through the annulus fibrosus (1, 2). The extruded nucleus pulposus can be associated with varying degrees of degenerative changes. In chondrodystrophic breeds such as the FBD the intervertebral disc degenerates via a process known as chondroid metaplasia. This involves significant changes within the nucleus pulposus including a loss of proteoglycans, a change from mainly collagen type II to collagen type I fibres, proliferation of chondrocyte-like cells and loss of notochordal cells, a dramatic loss of water content and usually significant calcification (3).

Magnetic resonance imaging (MRI) represents the gold standard diagnostic tool for IVDE (6–8). On MRI, compared to spinal cord parenchyma, extruded degenerated nucleus pulposus material usually appears as a hypointense mass within the epidural space on T1-weighted (T1w) and T2-weighted (T2w) images (8).

Intervertebral disc extrusion can also be associated with epidural haemorrhage, potentially causing further, multilevel spinal cord compression (9–11). Two recent retrospective studies provided evidence of the occurrence of epidural haemorrhage secondary to IVDE in small chondrodystrophic breeds such as FBD and dachshunds (10, 11). French bulldogs were reported to have a higher prevalence of epidural haemorrhage secondary to thoraco-lumbar IVDE (TL-IVDE) compared to Dachshunds and therefore potentially required more extensive surgical treatment than dogs without epidural haemorrhage to achieve spinal cord decompression (11). C-IVDE are reported to cause lesser neurological dysfunction than TL-IVDE (12). This was suspected to be due to a larger size of the vertebral canal in the cervical region compared to the thoracolumbar region. However, this assumption was not found to be true in FBD where the vertebral canal was relatively larger in the thoracolumbar region (13).

Thoracolumbar-IVDE in FBD have been described and compared to TL-IVDE in Dachshunds (14). To the authors’ knowledge, there is currently no study describing the clinical, MRI, surgical features of cervical IVDE (C-IVDE) and TL-IVDE in FBD. The aim of this study is to describe the clinical, MRI and surgical features of C-and TL-IVDE in FBD with surgically confirmed IVDE and to compare C-and TL-IVDE.

## Materials and methods

The medical records of a single United Kingdom-based referral centre (Southfields Veterinary Specialists, Linnaeus ltd) were retrospectively searched for FBD assessed for neurological disorders between 1st January 2020 and 1st March 2022. To fulfil inclusion criteria, FBD must have had neurological examinations at admission, discharge and 4-week post-surgery by a diplomate of the European College of Veterinary Neurology (ECVN) or an ECVN-resident working under direct supervision of an ECVN diplomate, an MRI confirming the diagnosis of IVDE (minimum requirement of T2w images in sagittal and transverse planes and T1w images in transverse plane) and decompressive spinal surgery.

Information retrieved from the medical records included: the patient’s signalment (age at diagnosis, sex, body weight), MRI diagnosis, surgical procedure performed, neurological grade at admission, at discharge and at reassessment 4 weeks post-treatment.

Based on the neurological assessment (including posture, gait, postural reactions, spinal reflexes and if required, nociception) lesions were localized to the C1-C5, C6-T2, T3-L3 or L4-S3 spinal cord segments (15). For all patients, neurological grade was evaluated using a six-point grading scale [adapted from Scott (16)]: grade 0 (neurologically unremarkable without hyperaesthesia), 1 (hyperaesthesia without neurological deficits), 2 (ambulatory paresis), 3 (non-ambulatory paresis), 4 (plegia with nociception) or 5 (plegia without nociception). Nociception was assessed by application of pressure to the digits and tail and assessing behavior response (13).

MR images of the vertebral column were acquired in all patients using a 1.5 Tesla MRI (Phillips Achieva 1.5 T system, Philipps medical system). Slice thickness ranged from 2 to 3 mm. Signal intensity was described relative to the signal of normal spinal cord parenchyma.

The following MRI features were recorded: IVDE location, longitudinal extent as a ratio of the extruded material length to the length of the C6 or L2 vertebral body (2, 17) presence and longitudinal extent of T2w increased intramedullary spinal cord signal intensity as a ratio of the intramedullary signal length to the length of C6 or L2 vertebral body (2, 17); degree of spinal cord compression classified as mild, moderate or severe based on modified morphologic scale (8), extruded IVD material signal intensity in T1w and T2w images. All MR images were reviewed by an ECVN diplomate and ECVN resident.

When degenerate EIVDM and blood were found concomitantly, haemorrhage was considered to be caused by IVDE (11). Any rupture of the venous sinuses that occurred during surgical treatment was not considered part of the bleeding caused by the IVD extrusion itself.

Improvement was considered if the patient presented an amelioration in its neurological grade by at least one point. Each patient was reassessed twice a day by a diplomate of the ECVN or by an ECVN-resident working under direct supervision of an ECVN diplomate. Time to improvement was obtained when an improvement was observed for the first time after spinal surgery. The outcome at 4-week reassessment was considered successful if the dog had improved in its neurological grade by at least one point and was comfortable (2). Comfort was appreciated via gentle palpation around the surgical site and evaluation of the response of the patient (13). The outcome at 4-week reassessment was considered unsuccessful if the neurological grade did not improve or deteriorated if the patient was euthanised or was still in discomfort.

## Statistical analysis

All variables collected were described by mean and range when normally distributed. Categorical variables were expressed as counts and percentages.

To compare all variables between the two groups (C-IVDE and TL-IVDE) Fisher’s Exact Test was used when data are categorical and Kruskal Wallis tests when data are scale (such as time) or ordinal (such as neurological grade) but unlikely to be normally distributed. The latter test is equivalent to the Mann Whitney test when, as in this case, there are only two groups.

Significance was assumed when *p* < 0.05.

## Results

**Figure 1:**
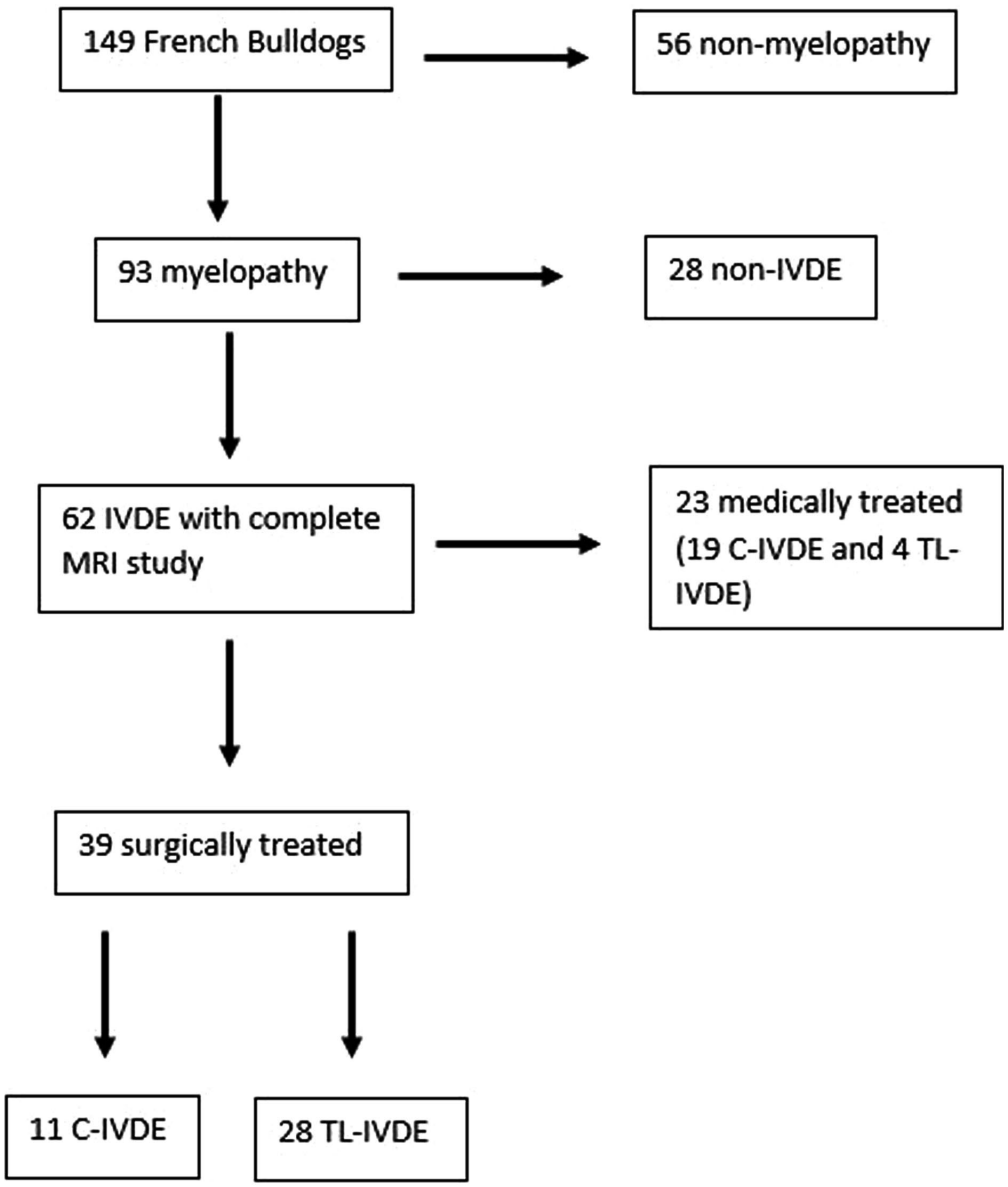
Flow diagram illustrating the inclusion and distribution of dogs in the study.

A total of 149 FBD were presented over the 26-month study period. Of these, 110 were excluded due lack of diagnostic investigations, due to diagnosis of a condition other than IVDE, due to medical management of IVDE or due to incomplete MRI study (Figure 1). Thirty-nine FBD met the inclusion criteria. The prevalence of MRI-confirmed IVDE in FBD was 65/149 (43%).

Fifteen FBD were female (6/15 spayed) and 24 were male (15/24 neutered). The mean age at diagnosis was 3.9 years (range 2.0–9.0 years). The mean body weight was 13.4 kg (range 8.3–20.0 kg). C-IVDE was diagnosed in 11/39 dogs (28%). TL-IVDE was diagnosed in 28/39 dogs (72%). There was no statistical association between age at diagnosis and location of the IVDE (*p* = 0.207) or between sex and location of the IVDE (*p* = 0.280).

**Figure 2:**
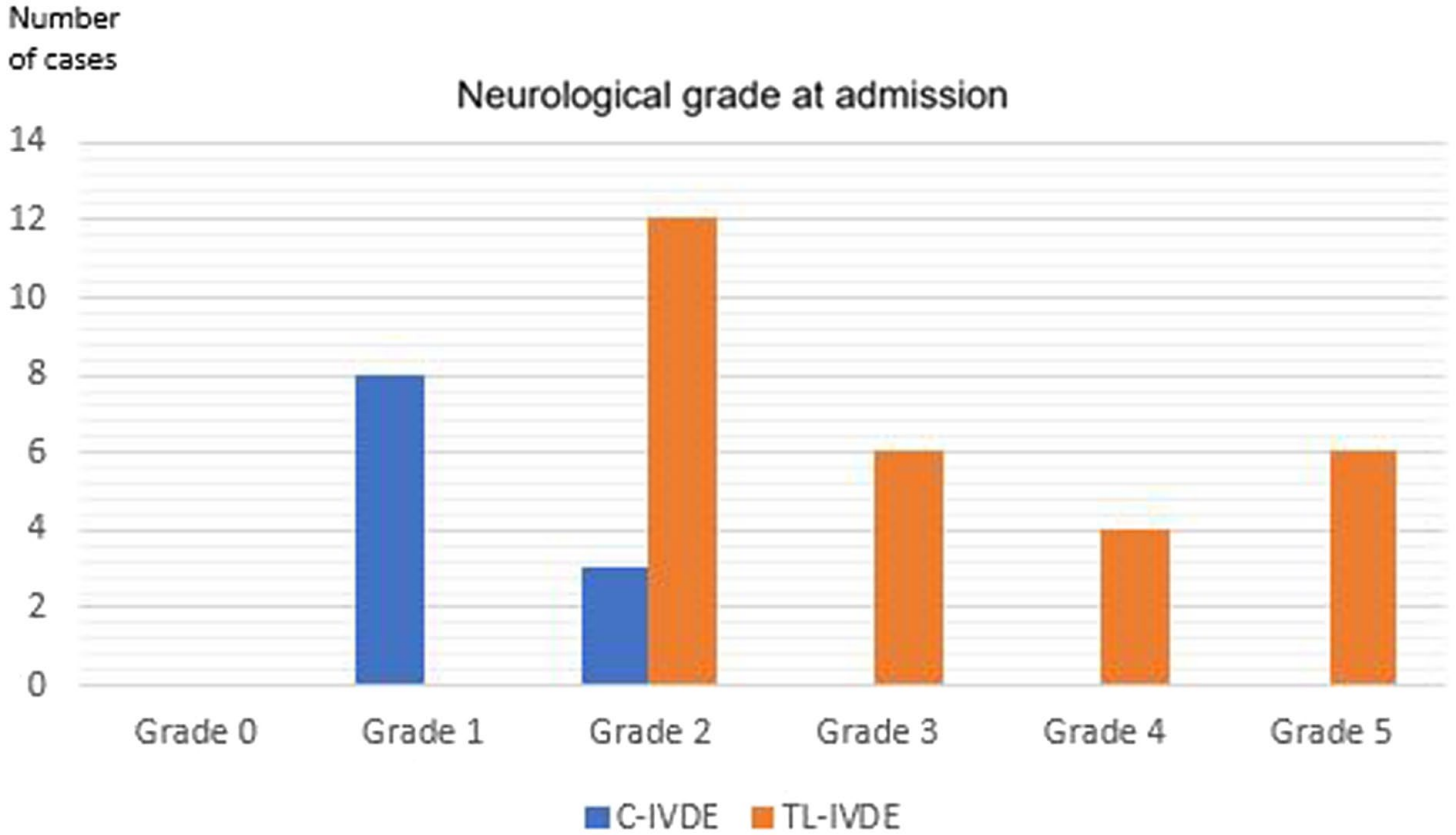
Neurological grade at admission.

Neurological grade at admission was grade 1 in 8/39 dogs (20.5%), grade 2 in 15/39 dogs (38.5%), grade 3 in 6/39 dogs (15.4%), grade 4 in 4/39 dogs (10.3%) and grade 5 in 6/39 dogs (15.4%). C-IVDE was significantly associated with less severe neurological grade (grade 1–2) compared to TL-IVDE (grade 2–5) at admission (*p* < 0.001) (Figure 2). The neuroanatomical localisation at admission was to the C1-C5 spinal cord segments in1/39 dogs (2.6%), to the C6-T2 spinal cord segments in 1/39 dogs (2.6%), to the T3-L3 spinal cord segments in 9/39 dogs (23.1%), L4-S1 spinal cord segments (19/39, 48.7%). Cervical hyperaesthesia without neurological deficits was observed in 8 C-IVDE at admission. One C-IVDE presented with a multifocal neuroanatomical localisation. This patient had received buprenorphine prior to referral.

**Figure 3:**
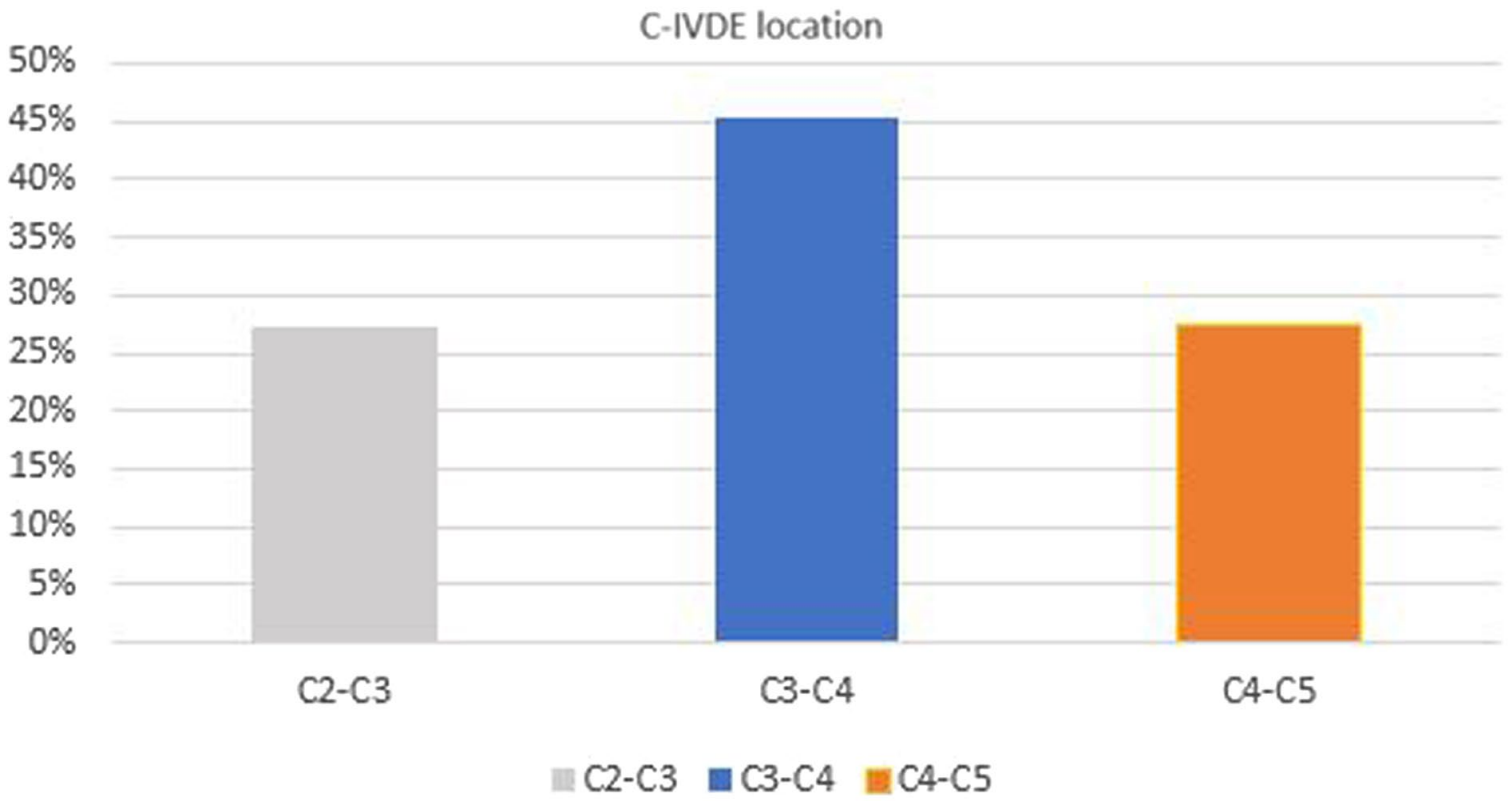
C-IVDE location.

**Figure 4:**
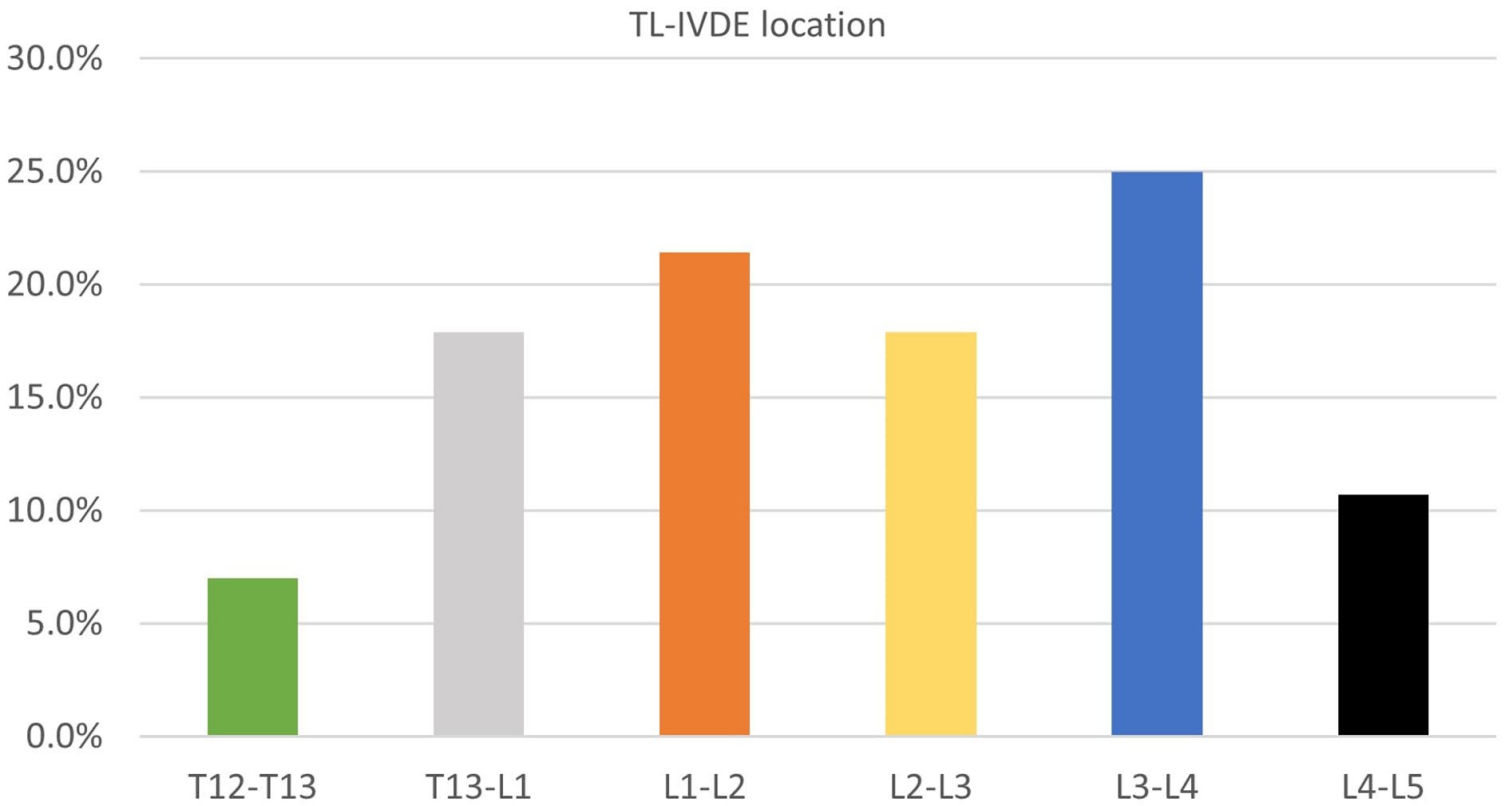
TL-IVDE location.

The offending IVD in C-IVDE was diagnosed at C3-C4 in 5/11 dogs (45.4%), C4-C5 in 3/11 dogs (27.3%), C2-C3 in 3/11 dogs (27.3%) (Figure 3). The offending IVD in TL-IVDE was diagnosed at L3-L4 in 7/28 dogs (25%), L1-L2 in 6/28 dogs (21.4%), T13-L1 in 5/28 dogs (17.9%), L2-L3 in 5/28 dogs (17.9%), L4-L5 in 3/28 dogs (10.7%) and T12-T13 in 2/28 dogs (7.1%) (Figure 4).

**Figure 5:**
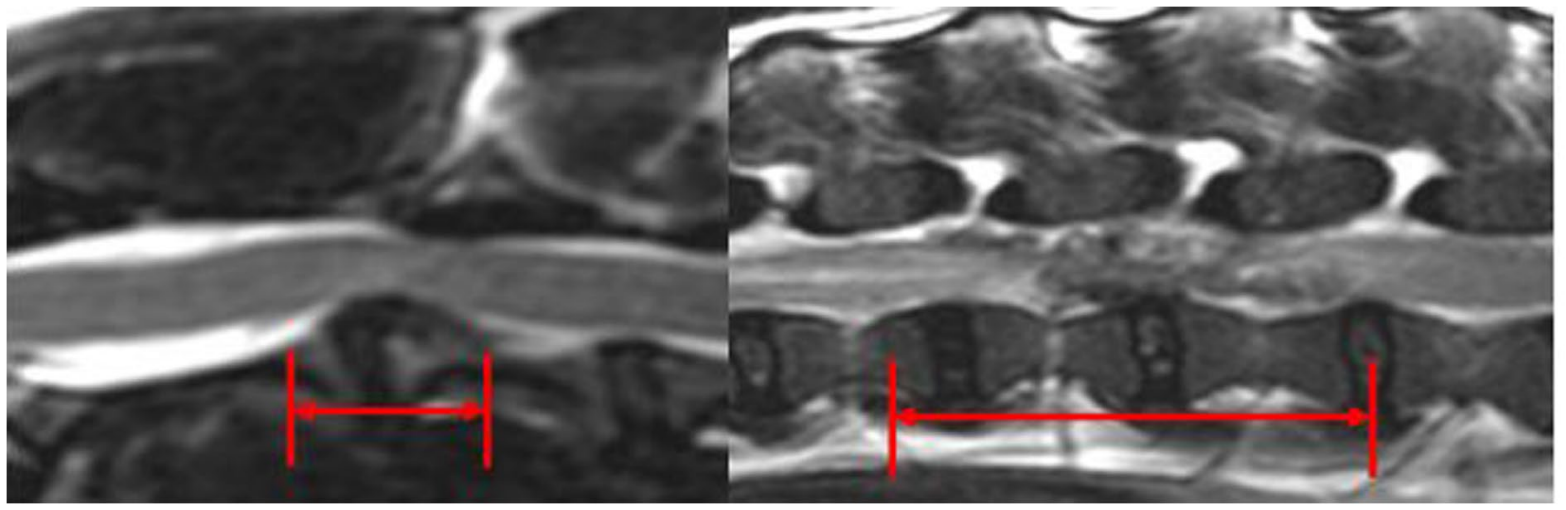
Mid-sagittal T2w images at the level of C2-C3 IVD (left) and L2-L3 IVD (right). The red arrows evaluate the longitudinal extent of the EIVDM within the vertebral canal.

Extruded IVDM was hypointense in T2w images in 11/11 C-IVDE and in 2/28 (7.1%) TL-IVDE, hypointense in T1w images in 10/11 C-IVDE (90.9%) and in 1/28 TL-IVDE (3.6%), hyperintense in T2w images in 0/11 C-IVDE and in 26/28 TL-IVDE (92.9%) and iso-to-hypointense in T1w images in 1/11 C-IVDE (9.1%) and in 27/28 TL-IVDE (96.4%) (*p* < 0.001). EIVDM extended ≥2 IVD spaces in 0/11 C-IVDE and in 19/28 TL-IVDE (67.9%) (*p* < 0.001). The mean longitudinal extent of the EIVDM was 0.9 (range 0.5–1.5) in C-IVDE and was 2.6 (range 0.3–4.6) in TL-IVDE (*p* < 0.001) (Figure 5).

T2w increased intramedullary spinal cord signal intensity was reported in 0/11 C-IVDE and in 24/28 TL-IVDE (85.7%) (*p* < 0.001). The mean longitudinal extent of the intramedullary T2w hyperintensity was 3.1 (range 1.0–5.6) in TL-IVDE. Spinal cord compression was mild in 2/11 C-IVDE (18.2%) and 3/28 TL-IVDE (10.7%); moderate in 9/11 C-IVDE (81.8%) and 16/28 TL-IVDE (57.1%); severe in 0/11 C-IVDE and 8/28 TL-IVDE (28.6%). There was no spinal cord compression in 1/28 TL-IVDE (3.6%). This dog was diagnosed with foraminal IVDE. There was no statistical association between the degree of spinal cord compression and IVDE location (*p* = 0.112).

All TL-IVDE (28/28) underwent unilateral hemilaminectomy whilst 10/11 C-IVDE (90.9%) underwent single ventral slot and 1/11 C-IVDE (9.1%) underwent a unilateral cervical hemilaminectomy. All C-IVDE and 9/28 TL-IVDE (32.2%) underwent a single space surgical treatment. No C-IVDE and 19/28 TL-IVDE (67.9%) underwent multiple IVD space surgery (*p* < 0.001). The mean number of IVD spaces surgically addressed was 2.07 IVD spaces (range from 1 to 4 IVD spaces) in TL-IVDE. No surgically related complications were found in any dog after surgical treatment. Haemorrhagic EIVDM was noticed in all cases where EIVDM was lateralised: 1/11 C-IVDE (9.1%) and 28/28 TL-IVDE (*p* < 0.001). These 29 cases had undergone a hemilaminectomy.

The overall mean duration of hospitalization post-surgical treatment was 2.7 days (range 1.0–8.0 days). The mean duration of hospitalization was 1.5 days in C-IVDE (range 1.0–3.0 days). The mean duration of hospitalization was 3.2 days in TL-IVDE (range 1.0–8.0 days). The overall mean time to improvement was 1.7 days (range 1.0–4.0 days). The mean time to improvement was 1.1 day in C-IVDE (range 1.0–2.0 days). 90.1% of C-IVDE improved within the first 24 h after surgical treatment. The mean time to improvement was 2.1 days in TL-IVDE (range 1.0–4.0 days).

**Figure 6:**
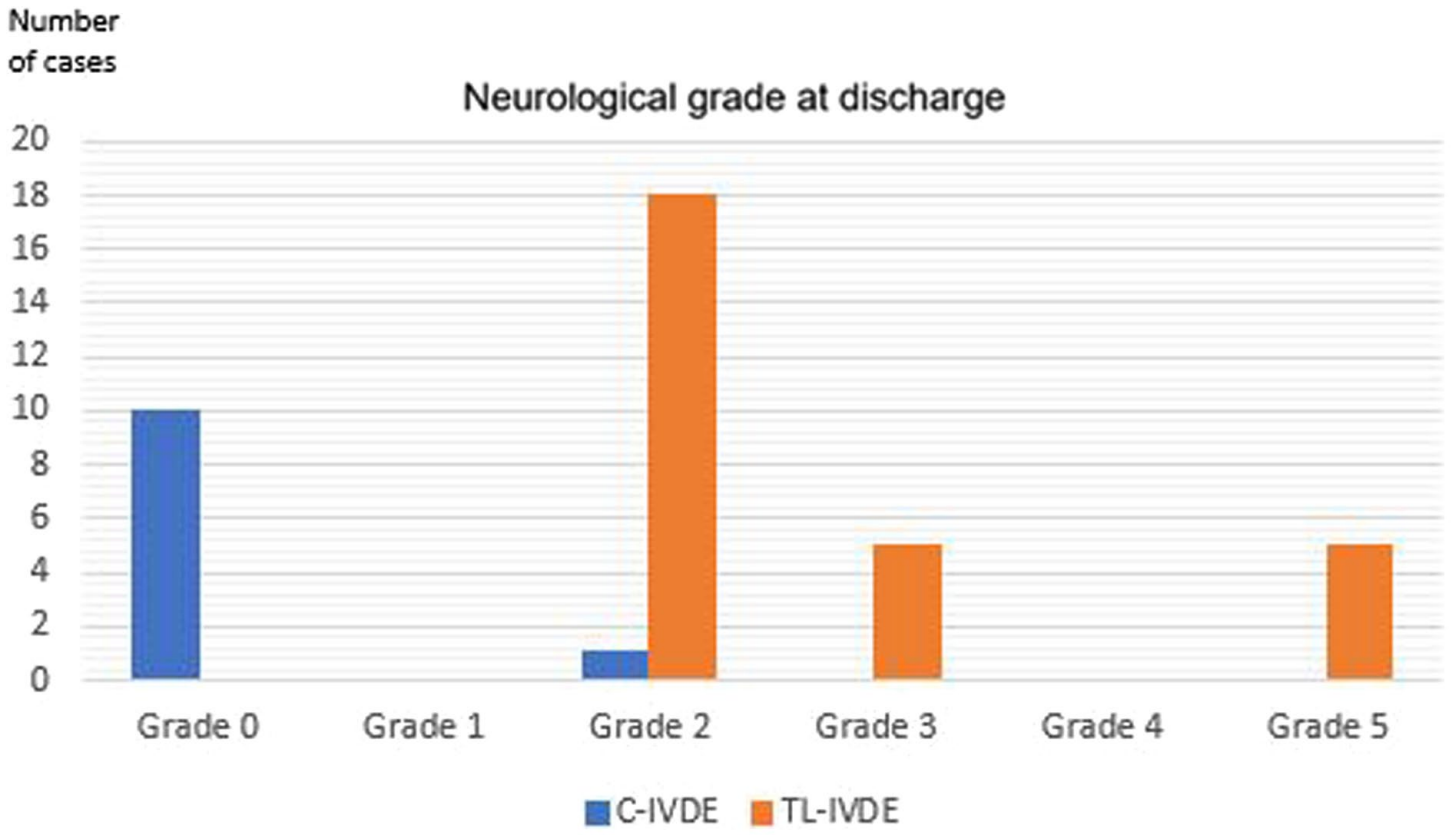
Neurological grade at discharge.

**Figure 7:**
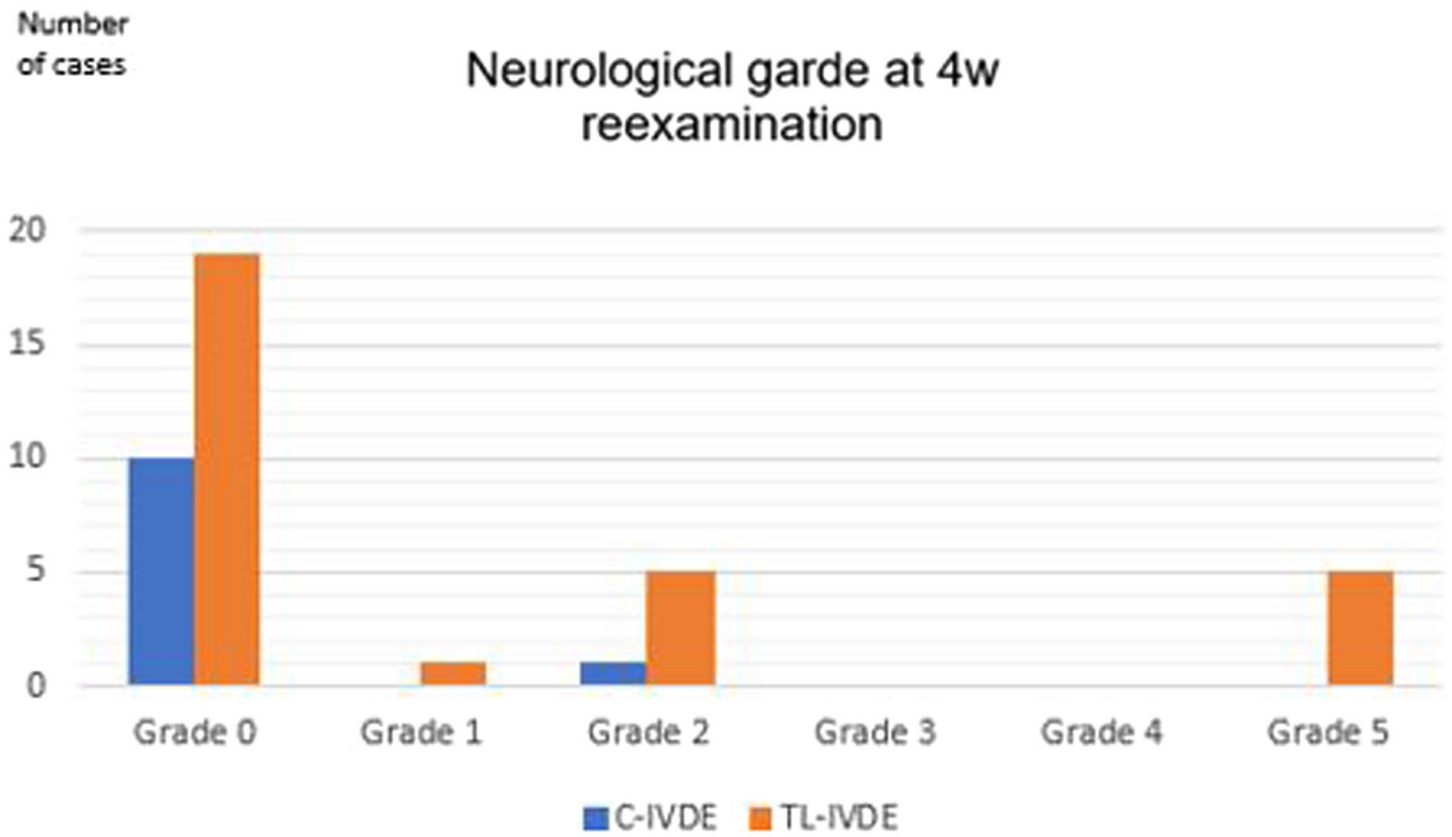
Neurological grade at 4 weeks reassessment.

Neurological grade at discharge was grade 0 in 10/39 dogs (25.6%), grade 2 in 19/39 dogs (48.7%), grade 3 in 5/39 dogs (12.8%) and grade 5 in 5/39 dogs (12.8%) (Figure 6). The neurological grade at discharge was significantly lower in the C-IVDE group compared to the TL-IVDE group (*p* < 0.001). Neurological grade at 4-week examination was grade 0 in 29/39 dogs (74.4%), grade 1 in 1/39 dogs (2.6%), grade 2 in 6/39 dogs (15.4%) and grade 5 in 5/39 dogs (12.8%) (Figure 7). The neurological grade at 4-week post-operative examination was not statistically different between C-IVDE and TL-IVDE (*p* = 0.063). Successful outcome at discharge was reported in 33/39 dogs (84.6%). Successful outcome at 4-week examination was reported in 34/39 dogs (87%). All C-IVDE had a successful outcome, whereas 23/28 TL-IVDE dogs (82.1%) had a successful outcome.

Unsuccessful outcome was reported in 5/39 dogs (12.8%). All were grade 5 TL-IVDE at admission and remained grade 5 at discharge and 4-week examination. They all presented with L4-S1 spinal cord segment neuroanatomical localisation at admission, discharge and 4-week examination. Only one dog had an IVDE location at L3-L4 IVD space potentially explaining the L4-S1 spinal cord segment neuroanatomical localisation. However, the remaining four presented IVDE at the level of T13-L1 (1), L1-L2 (2) and L2-L3 (1) IVD spaces. In these dogs, spinal shock was suspected. All grade 5 dogs that did not improve presented an increased extent of the EIVDM longitudinal extent (range 2.4–4.6), increased T2w intramedullary hyperintensity extent (Range 2.9–6.6). All but one underwent multiple IVD spaces hemilaminectomies. Haemorrhagic EIVDM was confirmed during surgical treatment in all cases. The duration of hospitalization of these dogs was longer than the mean duration of hospitalization in all but one of these dogs (range 7–8 days).

## Discussion

This retrospective study is the first to report the clinical, MRI and surgical features of C-IVDE and TL-IVDE in FBD. TL-IVDE presented with significantly higher grade of neurological dysfunction, required significantly more extensive surgical decompression and tended to require longer time to improvement than C-IVDE. The authors believe the present study could provide useful information for clinicians regarding MRI finding, surgical planning and prognosis for C-IVDE and TL-IVDE in FBD and help in their discussion with owners.

Based on our institution’s records, 98 FBD were presented for investigations of myelopathy. An MRI diagnosis of IVDE was obtained in 65/149 (43%) FBD presenting for neurological evaluation. The prevalence of IVDE in FBD in the present study is similar to previously reported in the literature (5). This result may have been higher as some of the FBD presented for investigations into myelopathy did not undergo investigations because of improvement on symptomatic treatment or cost-restrictions. A retrospective study reported the prevalence of neurological diseases in a large population of 343 FBD assessed in a single referral institution (5). The study revealed that 156 FBD (45.5%) were diagnosed with IVDE (5). Within the IVDE subgroup, thoracolumbar-IVDE was reported in 60.2% and C-IVDE in 39.8% of IVDEs (5). C3-C4 IVD space was the most common site for C-IVDE (5). In accordance with the latter, C-IVDE was most common at C3-C4 IVD space in the present study (5).

In the present study, TL-IVDE occurred more often within L1-L5 than T12-L1 IVD spaces (21/28 vs. 7/28 TL-IVDE respectively), with TL-IVDE most reported at L3-L4 IVD space. This finding is in agreement with another previous retrospective study comparing TL-IVDE in FBD and Dachshunds (14). That study reported a distribution of IVDE in the middle lumbar region in FBD compared to the thoracolumbar junction in Dachshunds (14). The cause of the difference of distribution of IVDE in FBD compared to Dachshunds remains unclear. Vertebral malformations have been suggested to cause biomechanical changes of the vertebral column (12). Aikawa et al. (14) evaluated the relationship between IVDE and vertebral malformations in FBD and observed that none of the FBD had IVDE within the kyphotic/kyphoscoliotic segment nor had a direct relationship between vertebral malalignment and IVDE location. In the kyphotic/kyphoscoliotic group of FBD, all IVDE occurred between 2 and 10 IVD spaces away from the kyphotic/kyphoscoliotic segment (14). The influence of vertebral malformations on the biomechanical changes on the vertebral column remain unclear and further studies evaluating their impact on adjacent IVD would be required. Evaluation of the influence of vertebral malformations on IVDE was outside the scope of this study; However, we did not identify an IVDE at an IVD space directly adjacent to a vertebral malformation.

Magnetic resonance imaging is the gold standard for the diagnosis of IVDE (6–8). Extradural degenerated extruded nucleus pulposus is typically defined as a hypointense mass within the epidural space in T1w and T2w images (18, 19). In our study, C-IVDE showed these characteristic features of IVDE. However, the EIVDM in TL-IVDE was either hypointense or hyperintense in T2w images and iso to hypointense in T1w images. T2w hyperintense EIVDM has been reported in hydrated nucleus pulposus IVDE, associated with various degrees of spinal cord compression (20). As the nucleus pulposus of FBD undergoes chondroid metaplasia, its MRI features would be of lower signal intensity in T2w images depending on the degree of degeneration (6–8). The mixed EIVDM intensities identified in our study was therefore suspected to be related to haemorrhagic EIVDM. Haemorrhage is associated with various signal intensities in T1w and T2w images (21, 22). Gradient echo MRI is highly sensitive for detection of blood products and detects chronic haemorrhage (23). Haemoglobin is strongly paramagnetic, leading to a low signal (signal void) in gradient echo images (23). As MRI characteristics of haemorrhage can be variable, intra-operative observation of haemorrhagic EIVDM was considered the standard to confirm haemorrhagic EIVDM. In the present study, the purpose of the MRI investigation was to diagnose IVDE. Therefore, T1w and T2w images were considered sufficient. To avoid unnecessarily extending the general anaesthesia of our patients, gradient echo sequences were not systematically performed in our cases.

In the present study, TL-IVDE cases had a significantly higher grade of neurological dysfunction than C-IVDE cases, with all C-IVDE being grade 1–2 and all TL-IVDE being grade 2–5. The degree of spinal cord compression was not significantly different between C-IVDE and TL-IVDE. However, the mean longitudinal extent of the EIVDM in TL-IVDE was significantly greater than in C-IVDE. Multilevel spinal cord compression could contribute to the greater neurological grade of TL-IVDE compared to C-IVDE. Multiple factors can contribute to the severity of the clinical signs in spinal cord injury (24). Primary spinal cord injury includes compressive and concussive injury (24). Secondary spinal cord injury refers to the molecular and biomechanical changes that occur following the primary injury (24, 25). Haemorrhage secondary to IVDE can cause severe spinal cord injury by contributing to the extradural compression (21, 22). Thoracolumbar-IVDE was significantly more likely to be associated with intramedullary T2w hyperintensity. Intramedullary hyperintensity identified on T2w images has been associated with necrosis, myelomalacia, intramedullary haemorrhage, inflammation, and oedema (8). Without differentiating the pathologic process more specifically, T2w hyperintensity has been shown to correlate well with the severity of neurologic signs at presentation in dogs with IVDE (18, 19, 26). The authors suspect that TL-IVDE severity compared to C-IVDE could be multifactorial, consisting of multilevel spinal cord compression by the EIVDM and its haemorrhagic component and severe spinal cord concussion at the moment of extrusion. Due to the longer longitudinal extent of EIVDM, higher prevalence of haemorrhagic-EIVDM, higher prevalence of T2w intramedullary hyperintensity in TL-IVDE compared to C-IVDE, TL-IVDE could occur in a higher energy-higher velocity context compared to C-IVDE.

Significant differences between the characteristics of IVDE in the cervical and thoracolumbar region were observed in the present study. The mean longitudinal extent of EIVDM in C-IVDE (0.9 times the length of C6 vertebra) was significantly shorter than the mean longitudinal extent of EIVDM in TL-IVDE (2.5 times the length of L2 vertebra). Extradural IVDM was therefore significantly less spread throughout the vertebral canal in C-IVDE than in TL-IVDE. The meningovertebral ligament is a ligamentous structure that extends from the ventral external surface of the dura mater to the dorsal aspect of the vertebral bodies and IVDs, forming a continuous attachment (27). In dogs, it is more robust in the cervical portion of the vertebral column, particularly between C3 and C5/C6 vertebrae and is finer and less robust in the thoracolumbar and lumbar regions (27). This anatomic barrier could contribute to avoid dispersion of the EIVDM in C-IVDE compared to TL-IVDE. Another hypothesis is that TL-IVDE occurred in a high energy-high velocity context, more easily tearing the meningovertebral ligament and spreading over multiple IVD spaces. In the present study, almost all C-IVDE (8/11) occurred between C3 and C5/C6 vertebrae and were all localized over the affected IVD space. The more robust meningovertebral ligament in this region could explain the more focal IVDE in the cervical region.

Owing to the significantly more widespread EIVDM, FBD with TL-IVDE more frequently underwent spinal surgery over multiple IVD spaces, compared to FBD with C-IVDE which more often underwent single space spinal surgery. Multi-IVD space surgical treatment in the TL region raises the question of biomechanical changes in the vertebral column (such as vertebral column instability). No major complications were recorded in patients undergoing multiple IVD space surgery. This is in accordance with another retrospective study on 23 cases of TL-IVDE with extensive epidural haemorrhage treated surgically (9). The authors of that study performed extensive hemilaminectomy surgery in all cases, including hemilaminectomies of up to five IVD spaces, without reporting any complications (9). The results of our study also suggested that surgically addressing multiple IVD spaces in the TL region does not seem to lead to complications. However, further biomechanical studies would be required to confirm that assertion.

Thoracolumbar-IVDE associated with macroscopic haemorrhagic-EIVDM has been documented in the veterinary literature under different nomenclature such as epidural haemorrhage and disc-associated epidural haemorrhage (DEEH) (9–12, 28). Epidural haemorrhage has been reported in 40% of dogs with TL-IVDE with FBD showing the highest prevalence of epidural haemorrhage (reported in 66% of TL-IVDE cases) (10). A retrospective study comparing occurrence of TL-IVDE disc-associated epidural haemorrhage between FBD and dachshunds reported a prevalence of DEEH in 41% of FBD and in 11% of dachshunds (11). The extruded nucleus pulposus can cause laceration of the internal vertebral venous plexus which lies on the floor of the vertebral canal (22). Computed tomography studies on the canine venous plexus in the thoracolumbar region revealed that the internal vertebral venous plexus was found to occupy the largest cross-sectional area relative to the vertebral canal cranially to the L1 vertebra (29, 30). French bulldogs are known for their marked variation from general anatomy such as vertebral malformation or vertebral body vascular dysplasia (14, 31). Vertebral body vascular dysplasia was not observed in the current study. Further anatomical studies on FBD may be required as haemorrhagic EIVDM could also be found in IVDE affecting IVD spaces caudal to L1 vertebra. The internal venous plexus vessels are the largest in the cervical region of dogs (32). Despite this feature, haemorrhagic EIVDM was only observed in one C-IVDE where the EIVDM was very lateralised. The remainder of C-IVDE FBD had EIVDM localized in the ventral midline. This patient was the only C-IVDE FBD to undergo a lateralised surgical approach. The cervical internal vertebral venous plexus is ventro-laterally positioned in relation to the spinal cord (30). This anatomical feature could explain the occurrence of haemorrhagic EIVDM in lateralised C-IVDE.

Despite more severe neurological grade at admission and at discharge, there was no statistical difference in the neurological grade between C-IVDE and TL-IVDE at 4-week examination. However, FBD presented with grade 5 neurological dysfunction tended to have a poorer prognosis of recovery. In the present study, the recovery rate at 4-week of grade 5 TL-IVDE FBD was 17%, which is lower than previously reported in patients with grade 5 neurological dysfunction (33). The only grade 5 TL-IVDE that had a successful outcome presented a rapid improvement and was discharged 2 days after surgical treatment with a neurological grade 3. All grade 5 TL-IVDE that did not improve had an L4-S1 spinal cord segment neuroanatomical localisation at admission, discharge and 4-week examination. This finding suggests that for grade 5 TL-IVDE FBD treated surgically, a rapid improvement within 24-48 h is a good prognostic indicator for successful recovery and that persistence of neurological deficits consistent with an L4-S1 spinal cord neuroanatomical localisation and absence of nociception is a poor prognostic indicator for recovery. However, a larger study would be required to determine whether these trends are statistically significant.

The present study had several limitations. The first limitation was related to its retrospective nature. Gradient echo MRI sequences were not in the inclusion criteria for this study. These sequences may have assisted in the detection of haemorrhage prior to surgical treatment. MRI may have not been able to differentiate between degenerated mineralized EIVDM and haemorrhagic EIVDM which would appear hypointense on T2w and gradient echo images (23).

No histological examination of the EIVDM was performed to confirm and grade the intensity of the haemorrhagic component. However macroscopic evaluation of EIVDM was considered sufficient to confirm the haemorrhagic component.

Another limitation is the difference in neurological grade at admission. The C-IVDE FBD presented with less severe neurological grade at admission. This less severe neurological grade at admission could influence some results such as the time to improvement and the time to discharge between the two groups, giving the trend for shorter time to improvement and shorter time to discharge for the C-IVDE group compared to the TL-IVDE group. A retrospective or prospective study comparing recovery time for similarly neurologically impaired C-and TL-IVDE FBD at admission may be required to confirm this trend.

A further limitation of this study is that follow-up was limited to 4 weeks. All but five patients had a successful outcome within the first 4 weeks post-surgical treatment. The five unsuccessfully treated patients were paraplegic without nociception at admission, discharge and 4-week reassessment. A longer post-operative follow-up may have been useful to determine if further improvement, or the development spontaneous motor function without nociception (such as ‘spinal walking’) occurred in those cases, though this was outside the scope of the study and would not have impacted our results (34).

In conclusion, for the FBD population included in this study, TL-IVDE caused higher grade of neurological dysfunction at diagnosis and resulted in compression of neural structures over multiple IVD spaces, requiring more extensive surgical treatment compared to C-IVDE. Haemorrhagic EIVDM was a characteristic feature of TL-IVDE and also occurred in a lateralised C-IVDE. The results of this study may also alert the clinician of the possible longer recovery time of TL-IVDE after surgical treatment compared to C-IVDE in FBD.

## Data availability statement

The original contributions presented in the study are included in the article/supplementary material, further inquiries can be directed to the corresponding author.

## Author contributions

FS conceived the project. GA and AU designed the project. GA and OM collected the data. GA prepared the manuscript. All authors were involved in manuscript revision and approval of the submitted manuscript.

## Funding

This study received funding from Linnaeus Veterinary Limited for the Open Access fees. The funder was not involved in the study design, collection, analysis, interpretation of data, the writing of this article or the decision to submit it for publication.

## Conflict of interest

GA, FS, OM, and AU are employed by Linnaeus Veterinary Limited.

## Publisher’s note

All claims expressed in this article are solely those of the authors and do not necessarily represent those of their affiliated organizations, or those of the publisher, the editors and the reviewers. Any product that may be evaluated in this article, or claim that may be made by its manufacturer, is not guaranteed or endorsed by the publisher.
